# Contrasting Temporal Responses of Saproxylic Beetles and Their Natural Enemies to a Catastrophic Hurricane Disturbance

**DOI:** 10.1002/ece3.74106

**Published:** 2026-07-26

**Authors:** Samuel Novais, E. Jacob Crístobal‐Pérez, Paul Hanson, Emmanuel Arriaga‐Varela, G. Wilson Fernandes, Mauricio Quesada

**Affiliations:** ^1^ Red de Interacciones Multitróficas Instituto de Ecología A.C Veracruz México; ^2^ Laboratorio Binacional de Análisis y Síntesis Ecológica Universidad Nacional Autónoma de México‐Universidad de Costa Rica Morelia México; ^3^ Laboratorio Nacional de Análisis y Síntesis Ecológica, Escuela Nacional de Estudios Superiores Unidad Morelia Universidad Nacional Autónoma de México Morelia Michoacán México; ^4^ Escuela de Biología Universidad de Costa Rica San Pedro Costa Rica; ^5^ Red de Biodiversidad y Sistemática Instituto de Ecología A.C Veracruz México; ^6^ Laboratório de Ecologia Evolutiva & Biodiversidade, Departamento de Genética, Ecologia & Evolução Universidade Federal de Minas Gerais Belo Horizonte MG Brazil; ^7^ Knowledge Center for Biodiversity Belo Horizonte MG Brazil

**Keywords:** dead wood, hurricane Patricia, parasitoids, predatory beetles, tri‐trophic interactions

## Abstract

Saproxylic beetles (dead wood‐dependent) are among the organisms that can benefit from the conditions after hurricane disturbances. These positive effects can propagate throughout the food chain, leading to an increase in populations of organisms at higher trophic levels. After the large‐scale windthrow caused by Category 5 Hurricane Patricia (October 2015) in a tropical dry forest of Jalisco, Mexico, we started an experimental two‐year (January 2016 and 2017) study assessing the temporal response of saproxylic beetles and their natural enemies (predatory beetles, and parasitoids) to this catastrophic disturbance. Insects were sampled from 148 freshly cut branches of *Spondias purpurea* (Anacardiaceae) (72 in 2016 and 76 in 2017), left in field for 45 days and then placed in emergence traps. Although the species number and individuals were higher in 2016 (133 species; 22,797 individuals) than in 2017 (82; 11,165), response timing to post‐hurricane conditions varied greatly among the most abundant saproxylic beetle families/subfamilies and natural enemy groups. Saproxylic beetles with short developmental times (e.g., Scolytinae, and small Bostrichidae) and parasitoids were more abundant in the sampling soon after the hurricane, while those with longer developmental times (e.g., Cerambycidae, large Bostrichidae, and Lyctinae) and predatory beetles (e.g., Histeridae) were more abundant in the following year. Competition and predation could have been the factors that regulated the populations of small saproxylic beetles and their associated parasitoids in the second sampling year. We conclude that life history traits and density‐dependent factors are important forces that mediate the temporal responses of saproxylic beetles to hurricane disturbances.

## Graphical abstract

Small saproxylic beetles and parasitoids peaked soon after the disturbance. Large saproxylic beetles and predatory beetles were more abundant in the next year. Life‐history traits and density‐dependence interactions shaped insect responses.

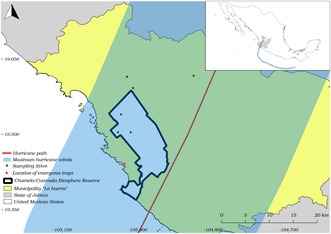

## 1 Introduction

Global climate change poses a significant and complex threat to biodiversity and the functions of ecosystems (IPCC 2023; Martén‐Rodríguez et al. 2025). Alongside elevated temperatures and modified precipitation patterns, climate change is causing an increase in the frequency, intensity, and duration of extreme weather events (Lange et al. 2020; Seneviratne et al. 2021). These events encompass temperature extremes, heavy precipitation and floods, droughts, and storms, including hurricanes/cyclones (Seneviratne et al. 2021). Particularly, the rise in extremely intense hurricanes (category 4 and 5 on the Saffir‐Simpson scale) observed in recent decades is likely associated with human‐induced climate change (Holland and Bruyère 2014; Sobel et al. 2016). Over the last four decades, the probability of hurricanes above category 3 has increased by 8% per decade (Kossin et al. 2020). In addition, hurricane occurrence projections in response to human‐induced warming have shown an increase in the frequency of high‐intensity hurricanes in numerous locations of the world (Knutson et al. 2020; Seneviratne et al. 2021). Considering the catastrophic potential of high‐intensity hurricanes for biological communities, it is essential to conduct studies that investigate the responses of different organisms to such disturbances.

Hurricanes play an important role by affecting the structure and dynamics of forest ecosystems (Connell 1978; Vandermeer et al. 1996; Eppinga and Pucko 2018; Parker et al. 2018). The main visible changes in forest structure caused by hurricanes are associated with the death or immediate reduction of vegetative and reproductive organs of many tree species, which may have contrasting impacts on other plants and associated animal communities (Lugo 2008; Schowalter et al. 2017; Novais et al. 2018, 2020). For instance, while epiphytic populations tend to decrease abruptly after hurricane events (Novais et al. 2020), the growth of pioneer plants is favored by an increase in sun exposure in the understory strata (Lugo 2008; Vandermeer et al. 1996). Among animals, several insect species may also benefit from an increase in the availability of different resources following hurricanes (e.g., foliage regrowth and increased dead wood), leading to increased populations (Bouget and Duelli 2004; Schowalter 2012; Novais et al. 2018).

Saproxylic insects, those that depend directly or indirectly on deadwood during part of their life cycle (Speight 1989), are among the organisms that can greatly benefit from post‐hurricane conditions (Bouget and Duelli 2004; Wermelinger et al. 2013; Novais et al. 2018). In addition to the large‐scale tree mortality, a large amount of fallen wood debris of different sizes and tree species represent key dead‐wood resources that enhance the diversity of saproxylic insect communities after hurricanes (Bouget and Duelli 2004). Among these insects, saproxylic beetles that directly feed on woody substrate (xylophagous), on cultivated fungi growing on branch cavities (i.e., mycetophagous), or on portions of wood and fungal tissue (i.e., xylomycetophagous) are expected to increase their populations following hurricanes (Gandhi et al. 2009; Wermelinger et al. 2013; Novais et al. 2018; Dodds et al. 2019; Miller et al. 2023).

The positive effects of hurricanes on saproxylic beetles at low‐order trophic levels can propagate throughout the food chain, causing an increase in populations of organisms at higher trophic levels, such as predatory beetles and parasitoids (Novais et al. 2018; Wermelinger et al. 2013). These natural enemies, in turn, contribute to the regulation of saproxylic beetle populations through trophic interactions (Bouget and Duelli 2004; Wermelinger et al. 2013). In addition to the top‐down control, populations of early‐colonizing saproxylic beetles tend to decline in abundance over the years as high‐quality dead wood resources (e.g., newly fallen and stressed trees) diminish (bottom‐up effect), indirectly affecting the populations of natural enemies at the third trophic level (Wermelinger et al. 2013). Therefore, a synchronous rise and fall between trophic levels is expected in the years following a hurricane disturbance (Novais et al. 2018, Wermelinger et al. 2013).

In October 2015, Hurricane Patricia, the strongest tropical hurricane (category 5) that has been reported in the Western Hemisphere so far, struck directly the tropical dry forest of the Chamela region in the state of Jalisco, Mexico. Nearly all trees were defoliated, stripped of their branches, snapped off, or uprooted by the strong winds (Kimberlain et al. 2015). We conducted a 2‐year experimental study to assess the temporal responses of saproxylic beetles and the incidence of natural enemies (predatory beetles and parasitoids) to this saproxylic beetle community after the hurricane. To assess the insect communities across years, we conducted a field experiment utilizing freshly cut branches (hereinafter referred to as “experimental branches”) of *Spondias purpurea* L. (Anacardiaceae), an abundant neotropical dioecious tree in the study region (Cristóbal‐Pérez et al. 2020). This tree species is the main host of the twig‐girdler beetle *Oncideres albomarginata chamela* (Chemsak and Giesbert) (Cerambycidae: Lamiinae), in which adult females girdle branches and make incisions along them for oviposition (Uribe‐Mú and Quesada 2006). Girdled and experimental branches provide suitable habitats for secondary colonization, mainly by saproxylic beetles that oviposit in the same branches (Calderón‐Cortés et al. 2011). Due to the ease of obtaining branches and their high abundance in the forest, the study of the saproxylic insect community associated with *S. purpurea* is a good model for understanding the response of these insects to a catastrophic hurricane disturbance. We hypothesize that an increase in dead wood availability following hurricane disturbances will rapidly favor growth of saproxylic beetle populations, which could lead to an increase in the populations of their natural enemies (bottom‐up effects). In addition, we hypothesize that the saproxylic beetle populations will be regulated through a decrease over time of dead wood resources (bottom‐up effects), and through predation pressure from higher trophic levels (top‐down effects). We predict that (1) the composition of saproxylic insects will differ between the period shortly after Hurricane Patricia devastated the study site (January 2016) compared to the following year (January 2017); (2) the temporal *β*‐diversity of saproxylic insects between the two sampling years will be primarily driven by species turnover; (3) the species richness and abundance of saproxylic beetles, and their natural enemies (predatory beetles and parasitoids) will be greater in 2016 compared to 2017; (4) the abundance within the most representative families/subfamilies of saproxylic beetles and predatory beetles will be greater in 2016 compared to 2017.

## 2 Material and Methods

### 2.1 Study Site

**FIGURE 1:**
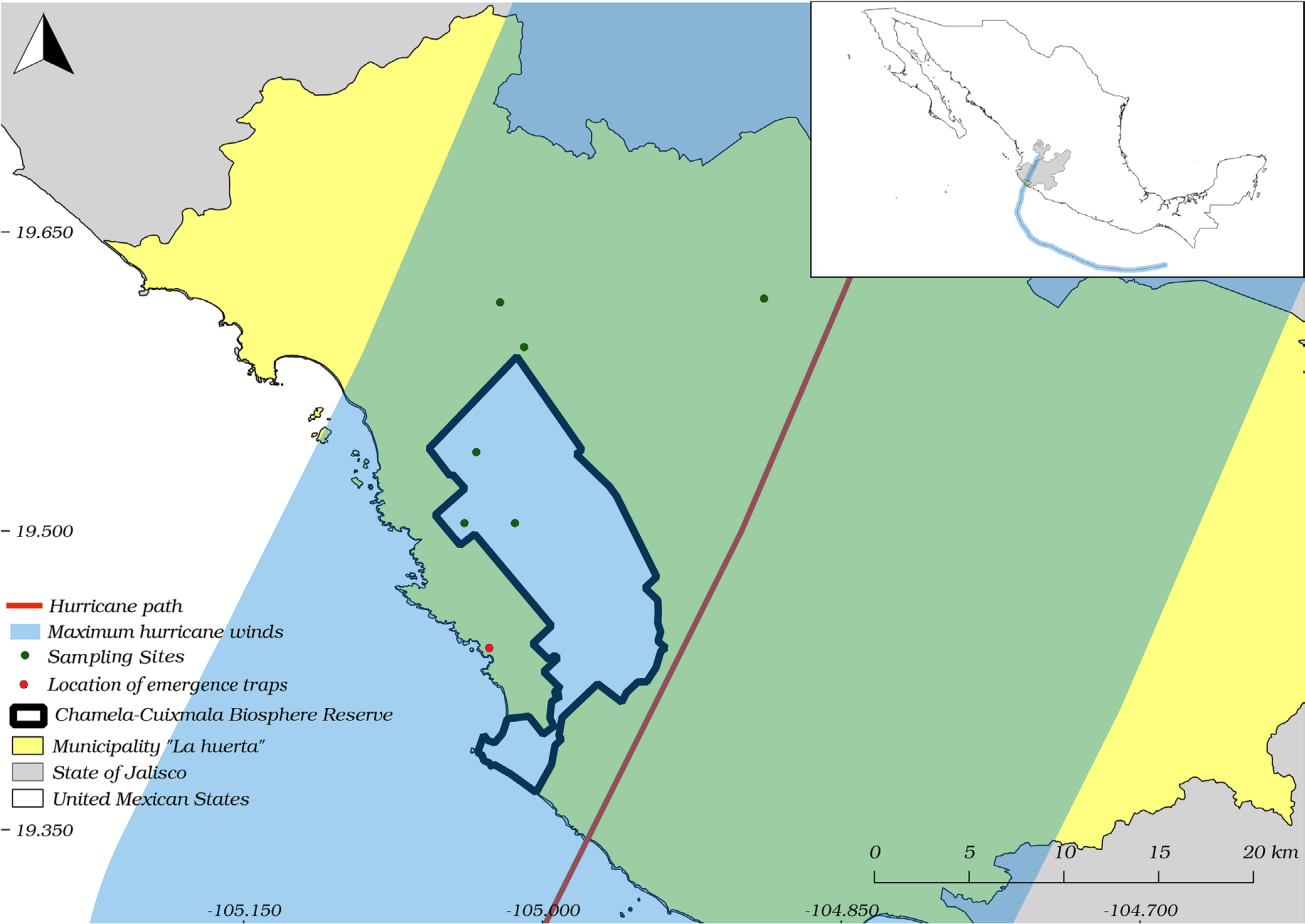
Map of the municipality of La Huerta, Jalisco, showing the location of the Chamela‐Cuixmala Biosphere Reserve and the six sampling sites. The red line and the blue boundaries represent respectively the path of Hurricane Patricia and their maximum winds obtained from historical hurricane tracks of the National Oceanic and Atmospheric Administration (https://coast.noaa.gov/hurricanes/).

This study was conducted in the municipality of La Huerta, located on the Pacific coast of Jalisco, Mexico (Figure 1). The region consists primarily of tropical dry forest with a mean annual rainfall of 748 mm and a marked dry season that extends from November to June (García‐Oliva et al. 2002). On the 23rd of October 2015, Hurricane Patricia, estimated as a category 5 on the Saffir‐Simpson Hurricane Wind Scale, reached the southwestern coast of Mexico in the state of Jalisco, heavily affecting the Chamela‐Cuixmala Biosphere Reserve (CCBR) region (Figure 1).

### 2.2 Experimental Design

Branches exhibiting similar characteristics (reproductive branches of ≈2–3 cm in diameter) to those detached and colonized by *Oncideres albomarginata chamela* were cut off from *Spondias purpurea* trees. These branches had an average length of 174 cm (SD±64 cm). To increase oviposition sites for saproxylic beetles, we simulated the structural modification of branches made by adult females of *O. albomarginata chamela* by making numerous incisions with scissors (every 5 mm) on the bark of the branches (see Calderón‐Cortés et al. 2011).

In January 2016, 3 months after Hurricane Patricia, six transects located at least 2 km apart were selected in the area where the hurricane's strongest winds impacted the region. (Figure 1). Three transects were selected within the CCBR, and another three transects were established in distinct forest fragments. Although differences between sites could represent a potential source of variation, transects were included as random effects to account for spatial heterogeneity (see Statistical analysis below). In each transect, we selected five individuals of *S. purpurea* (points) separated from each other by at least 50 m. Next, using a flagging tape, two or three experimental branches were placed on the understory vegetation under the canopy of the *S. purpurea* trees (*N* = 12 experimental branches/transect), for a total of 72 experimental branch samples in 2016. All experimental branches were marked and remained in the field for 45 days (January to February 2016) to allow the colonization by insects (Figure S1). After 45 days, the experimental branches were collected and enclosed individually in mesh bags (0.5 mm mesh size). Each bag was then placed inside an individual trap made of fine tulle fabric that allowed breathing but prevented further colonization and escape of the established fauna (Figure S2a). The emergence traps were placed in an out‐of‐service swimming pool and maintained under local environmental conditions (Figure S2b), near the Chamela‐Cuixmala Biosphere Reserve (Figure 1). The design of the emergence traps was similar to that of Winkler traps, with a 500 mL container with 300 mL of 70% ethanol attached to the bottom of the traps to collect and preserve specimens (Figure S2b). Emerging insects from each experimental branch were recorded monthly from the mesh bags and containers from March to December 2016 and were preserved in 70% ethanol until taxonomic identification. In January 2017, the same experiment was repeated using a new set of freshly cut *S. purpurea* experimental branches in the same transects and points. We used 76 experimental branches in 2017, for a total of 148 experimental branch samples during the 2‐year sampling period.

Taxonomic identification of beetles and parasitoids to family/subfamily level was carried out by E. Arriaga‐Varela and P. Hanson, respectively, using available taxonomic literature (e.g., Gauld 1997; Wharton et al. 1997; Arnett Jr. and Thomas 2001; Arnett Jr. et al. 2002). Some beetle species were identified to the genus or species level by comparison with specimens from a previous study (see Calderón‐Cortés et al. 2011). Beetles were divided into two groups regarding their trophic level: (1) Saproxylic beetles, those belonging to families/subfamilies with no predominant predatory feeding habit (e.g., xylophagous, mycetophagous, xylomycetophagous), and (2) Predatory beetles, those belonging to families/subfamilies with a predominant predatory/parasitoid feeding habit (Arnett Jr. and Thomas 2001; Arnett Jr. et al. 2002).

### 2.3 Statistical Analysis

The influence of year on the species composition of saproxylic insects at point scale (*N* = 30) was tested using permutational multivariate analysis of variances (PERMANOVA). We used the Bray–Curtis similarity measure and ran 999 permutations of residuals. To graphically illustrate differences in the assemblages of saproxylic insects we used nonmetric multidimensional scaling (NMDS). For NMDS, the ordination of species composition was also performed using the Bray–Curtis index and 999 permutations. A similarity percentage analysis (SIMPER) was performed to identify the species contributing most to the dissimilarity between years, based on Bray–Curtis distances (Clarke and Warwick 2001). We decomposed temporal *β*‐diversity to identify the primary mechanisms shaping the composition of saproxylic insects between years. *β*‐diversity was partitioned using the Sørensen (*β*_SØR_) and Simpson (*β*_SIM_) dissimilarity indices (Baselga 2010). *β*_SØR_ represents the total *β*‐diversity and includes both species turnover and nestedness. *β*_SIM_ reflects pure species turnover, independent of differences in species richness. The nestedness‐resultant component (*β*_NES_) was calculated as the difference between total dissimilarity and turnover (*β*_NES_ = *β*_SØR_—*β*_SIM_).

We used Generalized Linear Mixed Models (GLMMs; lme4 package in R) to test whether species richness (number of morphospecies per experimental branch) and abundance (number of individuals per experimental branch) of saproxylic beetles, predatory beetles, and parasitoids were affected by sampling years. Models were also performed separately for the abundance of the most representative families/subfamilies of beetles: Bostrichidae, Cerambycidae, Lyctinae (Bostrichidae), Histeridae, and Scolytinae (Curculionidae). We also fitted GLMMs to evaluate differences in the species richness and abundance of large beetles between sampling years. Species were classified as small or large based on their body length relative to the mean length of all species collected (mean 4.6 ± SE 0.4 cm). Species with a size smaller than 4.6 cm were considered small, while those with larger size were considered large. We estimated the mean size of each collected beetle species by measuring the length of the body of three individuals (when available) per species. The length of the beetles was measured from pictures using the software ImageJ (Schneider et al. 2012). In particular, Bostrichidae showed two general traits in terms of size: (a) species with small body size (hereafter referred to as “Bostrichidae small”), and (b) species with large body size (hereafter referred to as “Bostrichidae large”), which were analyzed by different models. Finally, we calculated the mean body size for each beetle family/subfamily (Table S1).

The models used (1) species richness and abundance (saproxylic beetles, predatory beetles, parasitoids, and large saproxylic beetles), and abundance within each of the most representative family/subfamily of saproxylic beetles as the response variables, (2) year as fixed explanatory variable, and (3) the transects as random effects. GLMMs and GLMs evaluating differences in overall insect species richness and abundance between years were compared through AIC to assess the variance explained by transect as a random effect. Including transect as a random effect substantially improved the fit of the models (species richness: ΔAIC = 16.4; abundance: ΔAIC = 11.9). We applied a “*Poisson*” distribution of errors to the species richness and abundance models, and overdispersion was adjusted with a “*Negative Binomial*” distribution of error. The *p*‐values of the fixed effect variables were estimated by likelihood ratio tests (ANOVA) between the models containing the predictor of interest and their respective null model (a simpler model without the predictor of interest) using a chi‐square test. To assess whether habitat type influenced the responses of saproxylic insects to hurricanes, we tested the interaction between year and habitat type (continuous forest vs. fragmented sites) for both overall species richness and abundance. The interaction was not significant for species richness (Poisson, LRT: χ^2^ = 2.71, *p* = 0.10) nor for abundance (Negative binomial, LRT: χ^2^ = 2.79, *p* = 0.09). These results indicate that the effects of the hurricane disturbance were consistent across habitat types. Although differences in forest structure and management history between habitat types may contribute to variation in community metrics, they did not alter the direction or significance of the main temporal patterns associated with hurricane disturbance. Therefore, habitat type and its interaction with year were not retained in the final models. All analyses were performed using R software (R Core Team 2024).

## 3 Results

A total of 161 morphospecies (96 saproxylic beetles, 20 predatory beetles, and 45 parasitoids) and 33,961 individuals (24,894 saproxylic beetles, 5692 predatory beetles, and 3375 parasitoids) emerged from the 148 experimental branches of *Spondias purpurea* in the two sampling years. For saproxylic beetles, Scolytinae (Curculionidae) was the most diverse group (16 spp.), followed by Cerambycidae (13), and Bostrichidae (small and large) (12; Table S1). Bostrichidae was the most abundant family/subfamily with 13,404 individuals (53.8% of all saproxylic beetles), followed by Scolytinae (Curculionidae) with 8312 (32.6%), representing together 98% of all saproxylic beetles (Table S1). For predatory beetles, Staphylinidae was the most diverse group (6 spp.), followed by Cleridae (5 spp.), while Histeridae was the most abundant family with 5608 individuals collected, representing 98.5% of all predatory beetles. For parasitoids, Braconidae was the most diverse parasitoid family (19 spp.), followed by Bethylidae (6 spp.) and Pteromalidae (5 spp.; Table S2). Braconidae was also the most abundant family with 3064 individuals (91% of all parasitoids), followed by Eurytomidae with 128 (4%), and Bethylidae with 82 (2%) (Table S2).

In 2016, twice as many insects were collected compared to 2017. A total of 22,796 individuals (18,305 saproxylic beetles, 1569 predatory beetles, and 2922 parasitoids) and 132 morphospecies (78 saproxylic beetles, 15 predatory beetles, and 38 parasitoids) were collected in 2016 compared to 11,165 individuals (6589 saproxylic beetles, 4123 predatory beetles, and 453 parasitoids) and 82 morphospecies in 2017 (43 saproxylic beetles, 13 predatory beetles, and 26 parasitoids). The most abundant species changed between the sampled years; while in 2016 there was the xylophagous beetle *Prostephanus truncates* (Horn) (Bostrichidae small), in 2017 the predatory beetle *Teretriosoma nigrescens* Lewis (Histeridae).

**FIGURE 2:**
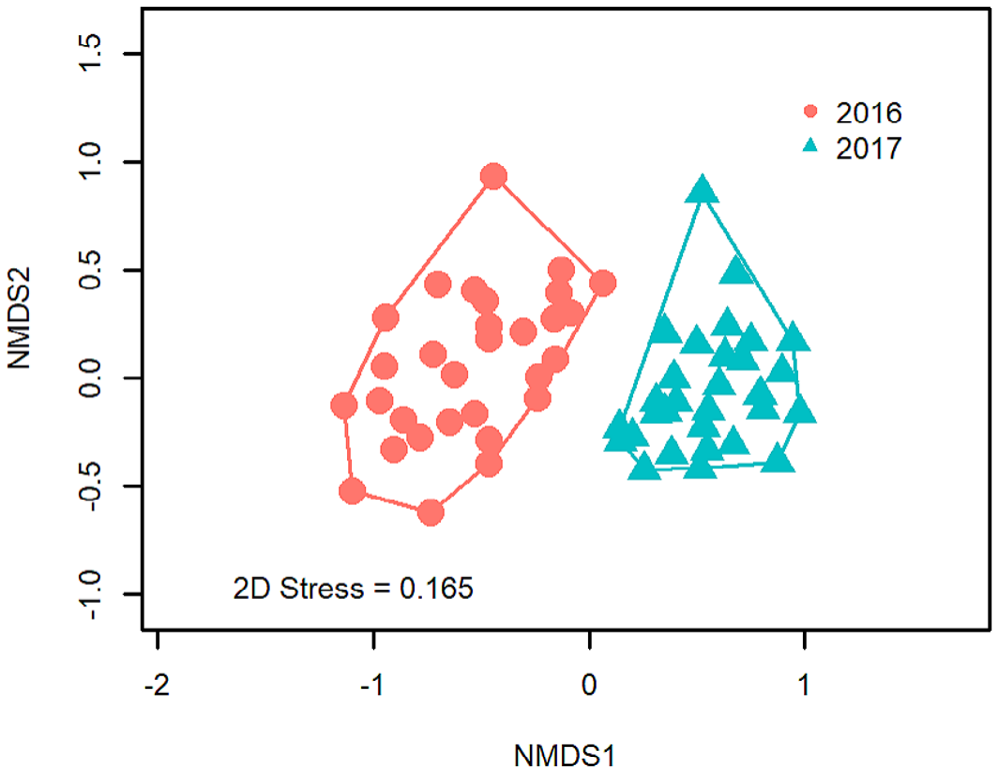
Nonmetric multidimensional scaling for the ordination of saproxylic insects that emerged from *Spondias purpurea* branches in a tropical dry forest, in Jalisco, Mexico, in 2016 and 2017. A significant difference in species composition between years was observed from PERMANOVA analyses (*p* = 0.001).

**FIGURE 3:**
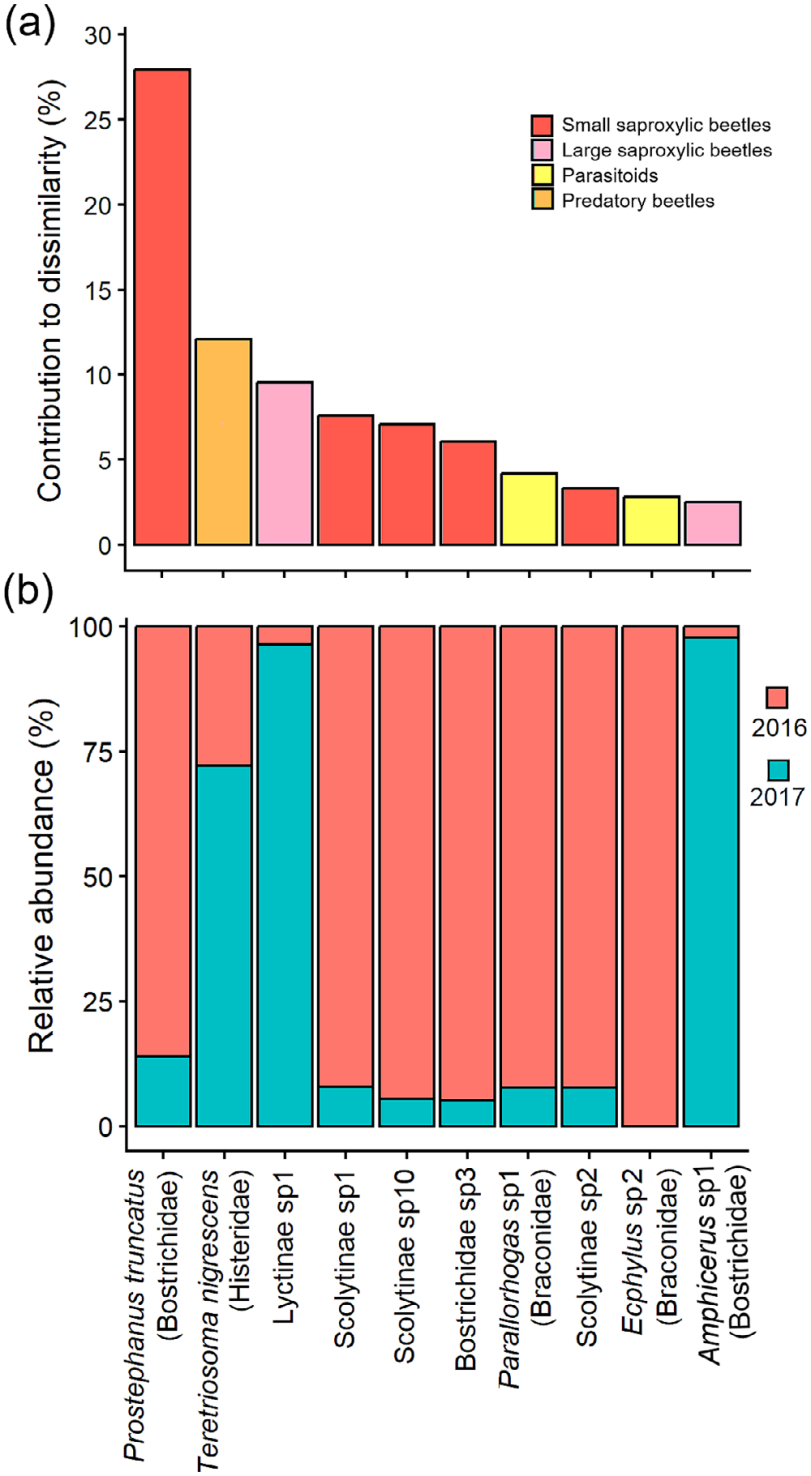
Relative contribution (%) of the ten most influential species to the dissimilarity between 2016 and 2017, as determined by a SIMPER analysis based on Bray–Curtis distances (a), and relative abundance (%) of the same species in 2016 and 2017 (b). Values in (b) were standardized within species to sum to 100%, allowing direct comparison of their relative contributions between years. Insects emerged from *Spondias purpurea* branches in a tropical dry forest, Mexico.

**FIGURE 4:**
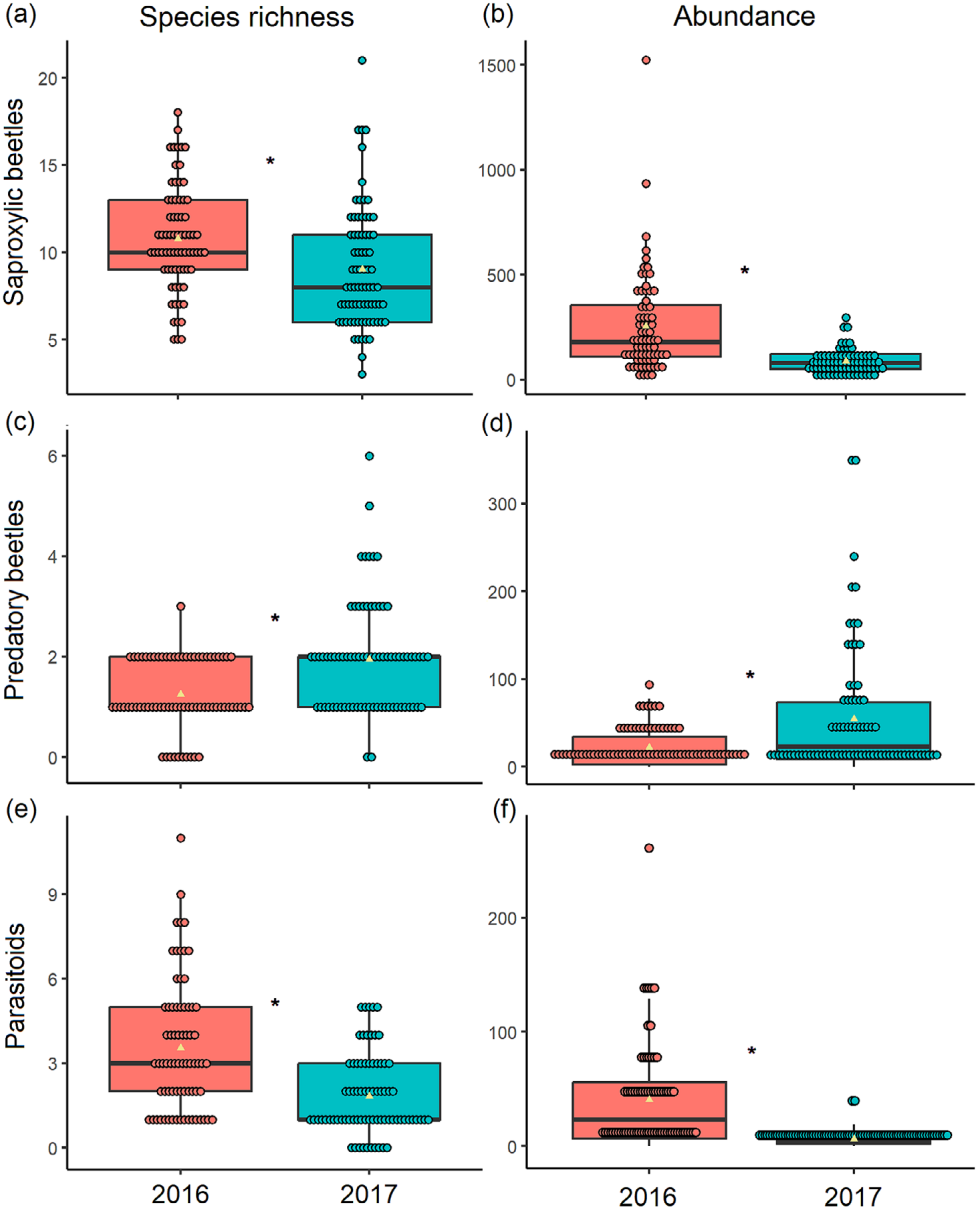
Boxplots of species richness (number of species/branch) and abundance (number of individuals/branch) of saproxylic beetles (a,b), predatory beetles (c,d), and parasitoids (e,f) between 2016 and 2017. Insects emerged from *Spondias purpurea* branches in a tropical dry forest, Mexico. Triangles represent mean values. Asterisks (*) represent significant differences among groups (GLMM, *p* < 0.05).

**FIGURE 5:**
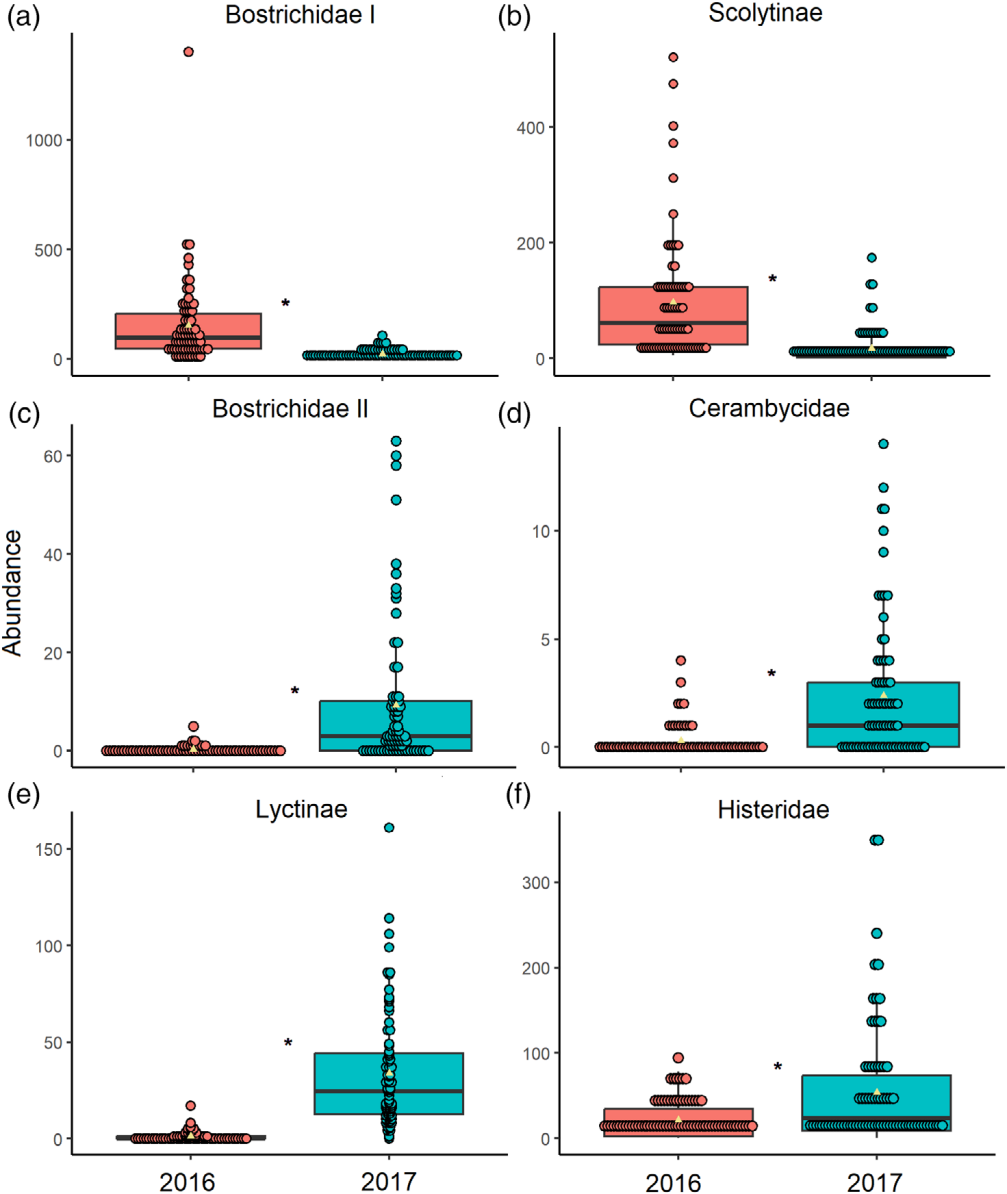
Boxplots of abundance (number of individuals/branch) of families/subfamilies of saproxylic beetles between 2016 and 2017. Bostrichidae small refers to species with small body size (< 4.6 mm), while Bostrichidae large refers to species with large body size (> 4.6 mm). Triangles represent mean values. Asterisks (*) represent significant differences among groups (GLMM, *p* < 0.05).

**FIGURE 6:**
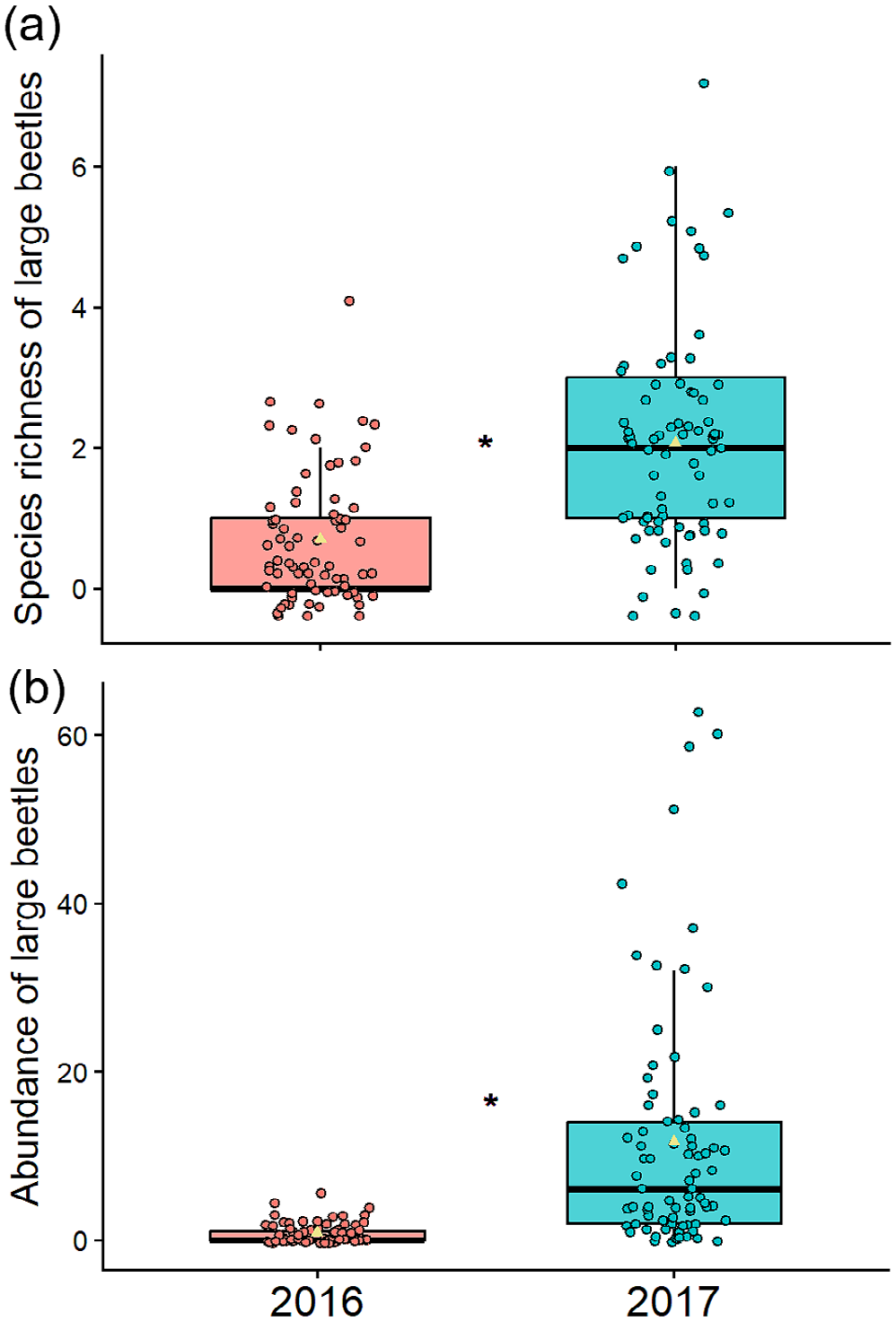
Boxplots of species richness (number of species/branch) (a) and abundance (number of individuals/branch) (b) of large saproxylic beetles between 2016 and 2017. Insects emerged from *Spondias purpurea* branches in a tropical dry forest, Mexico. Triangles represent mean values. Asterisks (*) represent significant differences among groups (GLMM, *p* < 0.05).

The composition of insects differed significantly between 2016 and 2017 (*p* = 0.001, R^2^ = 0.28, F_1,58_ = 22.18, Figure 2). The 10 most important species identified by SIMPER accounted for 83.2% of the total dissimilarity between years, with *P. truncates* being the main contributor (27.9%), followed by *T. nigrescens* (12.1%), Scolytinae sp1 (9.6%), Lyctinae sp1 (7.6), Scolytinae sp10 (7.1%) (Figure 3a). In addition, the 10 most important species exhibited marked differences in relative abundance between years, with values ranging from 72% to 100% in a single year (Figure 3b). Partitioning of temporal beta diversity (*β*_SOR_ = 0.51) between years revealed species turnover as the main driver (*β*_SIM_ = 0.365), whereas nestedness contributed less (*β*_Nes_ = 0.145). Species richness and abundance of saproxylic beetles were significantly greater in 2016 compared with 2017 (χ^2^_df=1_ = 11.094, *p* < 0.001; χ^2^_df=1_ = 59.602, *p* < 0.0001, respectively) (Figure 4a,b). Natural enemies presented contrasting results; while a significantly greater species richness and abundance of parasitoids were found in 2016 compared with 2017 (χ^2^_df=1_ = 39.981, *p* < 0.0001; χ^2^_df=1_ = 69.884, *p* < 0.0001, respectively) (Figure 4c,d), the opposite pattern for predatory beetles (χ^2^_df=1_ = 11.316, *p* < 0.001; χ^2^_df=1_ = 17.709, *p* < 0.0001, respectively) (Figure 4e,f). The analysis conducted separately for the most abundant saproxylic beetle families/subfamilies also demonstrated contrasting results. Bostrichidae small and Scolytinae were significantly more abundant in 2016 (χ^2^_df=1_ = 101.79, *p* < 0.0001; χ^2^_df=1_ = 95.413, *p* < 0.0001, respectively) (Figure 5a,b), while Bostrichidae large, Cerambycidae, Lyctinae, and Histeridae in 2017 (χ^2^_df=1_ = 67.26, *p* < 0.0001; χ^2^_df=1_ = 42.075, *p* < 0.0001; χ^2^_df=1_ = 154.06, *p* < 0.0001, χ^2^_df=1_ = 16.409, *p* < 0.0001, respectively) (Figure 5c–f). Species richness and abundance of large saproxylic beetles were significantly greater in 2016 compared with 2017 (χ^2^_df=1_ = 54.05, *p* < 0.001; χ^2^_df=1_ = 89.421, *p* < 0.0001, respectively) (Figure 6a,b).

## 4 Discussion

The community structure of saproxylic beetles and their natural enemies changed dramatically in the 2 years following Hurricane Patricia. In accordance with our expectations, the composition of insects differed significantly between 2016 and 2017, with species turnover being the main driver of temporal beta diversity. In addition, a greater species richness and abundance of saproxylic beetles were found soon after the hurricane compared to the next year. Contrasting results were observed for natural enemies, with parasitoids being more species‐rich and abundant in 2016, while predatory beetles in 2017. Finally, the response time to post‐hurricane conditions varied greatly among the most abundant beetle families/subfamilies. While some families/subfamilies were more abundant in the second sampling year (i.e., Bostrichidae large, Cerambycidae, Lyctinae, and Histeridae), others were soon after the hurricane disturbance (Bostrichidae small and Scolytinae).

In our study, a general increase in early‐colonizing saproxylic insect populations was expected soon after the hurricane due to the increased amount and diversity of high‐quality deadwood resources (e.g., newly fallen branches), which would lead to a greater colonization of experimental branches by saproxylic beetles and their natural enemies in 2016 compared to 2017. Although we did not quantify deadwood availability after the hurricane, Parker et al. (2018) reported a 23% loss of aboveground biomass following Hurricane Patricia in the same region. The expected pattern was clearly observed for the total number of families/subfamilies recorded between years: 33 of the 35 taxa were collected in 2016 compared to only 17 in the second sampling year (Table S1). On the other hand, the overall decrease in species richness and abundance in 2017 was driven mainly by Scolytinae and Bostrichidae small, which together represented 84% of all sampled saproxylic beetles. Both groups present small body sizes and are considered early‐decay species, breeding mainly in the phloem or xylem of freshly dead wood (Wood 1982). In addition, they present short developmental times, with the total life‐span of most species being lower than 3 months (Wood 1982). In the second sampling year, Scolytinae and Bostrichidae small probably had to deal with greater interspecific competition with species from groups with a longer development time and/or large body size in experimental branches. For example, the Lyctinae subfamily, the third most abundant group of saproxylic beetles (10.7% of total), was 28 times more abundant in 2017 than 2016. Despite their small body size (Table S1), members of this subfamily have longer development times compared to Bostrichidae small and Scolytinae, ordinarily requiring about nine to 12 months to complete their life cycle (from egg to adult) (Gerberg 2008; Haverty 2003). These beetles probably took longer to increase their populations, being more abundant in the second year.

In addition to the Lyctinae subfamily, two families of large‐bodied saproxylic beetles, Bostrichidae large and Cerambycidae, were 40 and 8.3 times more abundant in 2017 compared to 2016, respectively. A similar result was found by a previous study conducted in pine plantations in the United States, which evaluated the temporal responses of wood‐boring beetles (Buprestidae and Cerambycidae) to Hurricane Michael (category 5) by comparing samples taken seven and 20 months (2019 and 2020, respectively) after the landfall (Miller et al. 2023). The authors found that of the 32 species collected, 20 were more abundant in the second sampling year, with the total beetle abundance higher by 28.6% in 2020 than in 2019. Dodds et al. (2019) also found a higher abundance of Cerambycidae beetles in the second year of sampling after an EF1 tornado damaged coniferous forests in Maine, USA. The authors highlighted that generation time was an important factor influencing this result, as some cerambycids take more than a year to develop. In the present study, the time since Hurricane Patricia and our first sampling (approx. 4 months) was probably not long enough for species with large body sizes to increase their population sizes, and it was not until the second year that these groups were able to colonize *S. purpurea* branches in large numbers. At this moment, the presence of these large beetle species has probably imposed strong competition for feeding and breeding sites with small early‐decay species in the branches and maintaining their populations at lower levels.

In addition to greater competition with other groups, Bostrichidae small and Scolytinae species probably also suffered greater predation pressure in the second year. In our study, predatory beetles were dominated by the family Histeridae, which was 2.7 times more abundant in 2017 than 2016. Histeridae was the most abundant group of beetles in the second year, dominated by the predator *Teretriosoma nigrescens*, with more emerging individuals than any family/subfamily of saproxylic beetles. A previous experimental study showed that *T. nigrescens* can suppress populations of *Prostephanus truncates* (Bostrichidae small), the most abundant saproxylic beetle in our study in 2016, with populations increasing by at least l0‐fold in treatments in the absence of this predator (Rees 1985). On the other hand, parasitoid species richness and abundance showed a similar pattern between years to that found for Bostrichidae small and Scolytinae, which suggests these two taxa are the main hosts of the parasitoids, which exercised a top‐down control over their populations in the first sampling year. A previous study conducted in subalpine spruce forests in the Swiss Pre‐Alps after Hurricane Vivian (1990) demonstrated that the populations of bark beetles (Scolytinae) and their associated parasitoids increased and declined rather synchronously after the disturbance (Wermelinger et al. 2013). The authors argue that these beetles took advantage of the increased amount of breeding resources in fresh dead wood following the hurricane and produced many offspring, which immediately served as hosts for the parasitoids. The subsequent decline of both host beetles and parasitoid populations 4 years after the hurricane was mainly determined by a reduction in availability of quality dead wood resources for beetles (Wermelinger et al. 2013). The present study suggests that a decrease in parasitoid populations was indirectly caused by the increase of large‐bodied saproxylic beetles and predators in the second year following Hurricane Patricia, leading to a decline in the populations of their primary hosts.

A limitation of this study is the absence of pre‐disturbance baseline data and the relatively short temporal scope (2 years), which constrain our ability to define baseline community conditions and to disentangle hurricane‐driven effects from natural interannual variability or stochastic colonization. Moreover, both sampling periods likely fall within the broader successional trajectory following the severe hurricane disturbance and may therefore represent different stages of post‐hurricane recovery rather than a return to pre‐disturbance conditions (see Novais et al. 2018). Although our results are consistent with mechanisms such as competition, predation, bottom‐up and top‐down dynamics, these processes were not directly tested. Further research incorporating experimental manipulations and longer‐term datasets is needed to explicitly evaluate the role of these mechanisms in shaping post‐disturbance community dynamics (Schowalter et al. 2017). Overall, our results should be interpreted as reflecting short‐term community dynamics during early recovery rather than as a comprehensive assessment of hurricane impacts on saproxylic beetles and their natural enemies.

## 5 Conclusions

Our findings highlight how severe density‐independent disturbances can restructure communities through shifts in resource availability and species life‐history traits, leading to temporally dynamic interactions between bottom‐up and top‐down processes during early recovery. These temporal shifts in species composition and interactions (e.g., competition and predation) may extend to other disturbance‐prone ecosystems, where resource pulses following large‐scale events can differentially favor species depending on their traits (Moretti et al. 2010; Batchudur et al. 2026). In particular, if the frequency and intensity of hurricanes increase under climate change scenarios, such dynamics could alter the timing and trajectory of community recovery, potentially favoring fast‐colonizing species and modifying trophic interactions across multiple taxa (Schowalter et al. 2017). Understanding how resource availability, life‐history traits, and trophic interactions shape community dynamics after disturbance is important for predicting how ecological communities respond to increasingly frequent and intense disturbance events. Importantly, our results also underscore the need to sample communities at multiple time points following disturbance events, as single or short‐term assessments may overlook key shifts in species composition and trophic interactions, leading to an incomplete understanding of disturbance effects on ecological communities.

## Author Contributions

**Samuel Novais:** conceptualization (equal), formal analysis (lead), investigation (lead), visualization (equal), writing – original draft (lead), writing – review and editing (equal). **E. Jacob Crístobal‐Pérez:** investigation (supporting), writing – review and editing (equal). **Paul Hanson:** investigation (equal), writing – review and editing (equal). **Emmanuel Arriaga‐Varela:** investigation (equal), writing – review and editing (equal). **G. Wilson Fernandes:** writing – review and editing (equal). **Mauricio Quesada:** conceptualization (equal), formal analysis (equal), funding acquisition (equal), supervision (equal), writing – review and editing (lead).

## Funding

This work was supported by Universidad Nacional Autónoma de México (PAPIIT IN219021, IN225924, IN224920, IN226423), Secretaria de Ciencia, Humanidades, Tecnologia e Innovacion (SECIHTI) to LANASE (LN2025‐C‐127), LANASE‐CIC‐UNAM 2015–2025, Proyecto Laboratorio Binacional de Análisis y Síntesis Ecológica UNAM‐UCR. S. Novais thanks CAPES (PDSE‐Sandwich Doctoral Programme Abroad) for research grants. E Jacob Cristobal‐Pérez was supported by SECIHTI (Postdoctoral fellowship 2022–2026, #3725518). GWF thanks CNPq, Fapemig and the Knowledge Center for Biodiversity for different grant support.

## Conflicts of Interest

The authors declare no conflicts of interest.

## Supporting information


**Figure S1:** Example of a *Spondias purpurea* branch exposed to colonization by saproxylic beetles and their natural enemies in the understory of a tropical dry forest, in Jalisco, Mexico.
**Figure S2:** A branch of *Spondias purpurea* double‐enclosed in mesh bags and emergence tulle traps (a). The traps were placed in an out‐of‐service swimming pool and maintained under local environmental conditions (b).
**Table S1:** Morphospecies richness (Rich.) and abundance (Abun.) of saproxylic beetles and predatory beetles that emerged from *Spondias purpurea* branches in a tropical dry forest, in Jalisco, Mexico, during two sampling years. Bostrichidae I refers to species with small body size (< 4,6 mm), while Bostrichidae II refers to species with large body size (> 4,6 mm). * Families of beetles considered predatory.
**Table S2:** Number of individuals of parasitoid morphospecies that emerged from *Spondias purpurea* branches in a tropical dry forest, in Jalisco, Mexico, during two sampling years.

## Data Availability

Data (DATASET: Saproxylic Insects) and R script are available in Figshare at https://figshare.com/s/a781c402bbba68a4654d.
