# Supplementary material for: Contrasting Temporal Responses of Saproxylic Beetles and Their Natural Enemies to a Catastrophic Hurricane Disturbance
Source: Ecol Evol. 2026 Jul 26;16(7):e74106. doi: 10.1002/ece3.74106 (PMC13402101; doi:10.1002/ece3.74106)
Supplement: Supplementary file 1 — Figure S1: Example of a Spondias purpurea branch exposed to colonization by saproxylic beetles and their natural enemies in the understory of a tropical dry forest, in Jalisco, Mexico. Figure S2: A branch of Spondias purpurea double‐enclosed in mesh bags and emergence tulle traps (a). The traps were placed in an out‐of‐service swimming pool and maintained under local environmental conditions (b). Table S1: Morphospecies richness (Rich.) and abundance (Abun.) of saproxylic beetles and predatory beetles that emerged from Spondias purpurea branches in a tropical dry forest, in Jalisco, Mexico, during two sampling years. Bostrichidae I refers to species with small body size (< 4,6 mm), while Bostrichidae II refers to species with large body size (> 4,6 mm). * Families of beetles considered predatory. Table S2: Number of individuals of parasitoid morphospecies that emerged from Spondias purpurea branches in a tropical dry forest, in Jalisco, Mexico, during two sampling years. [file ECE3-16-e74106-s001.docx]

**Supplemental Material for the manuscript:**

**“Contrasting temporal responses of saproxylic beetles and their natural enemies to a catastrophic hurricane disturbance”**

Contents

Figures


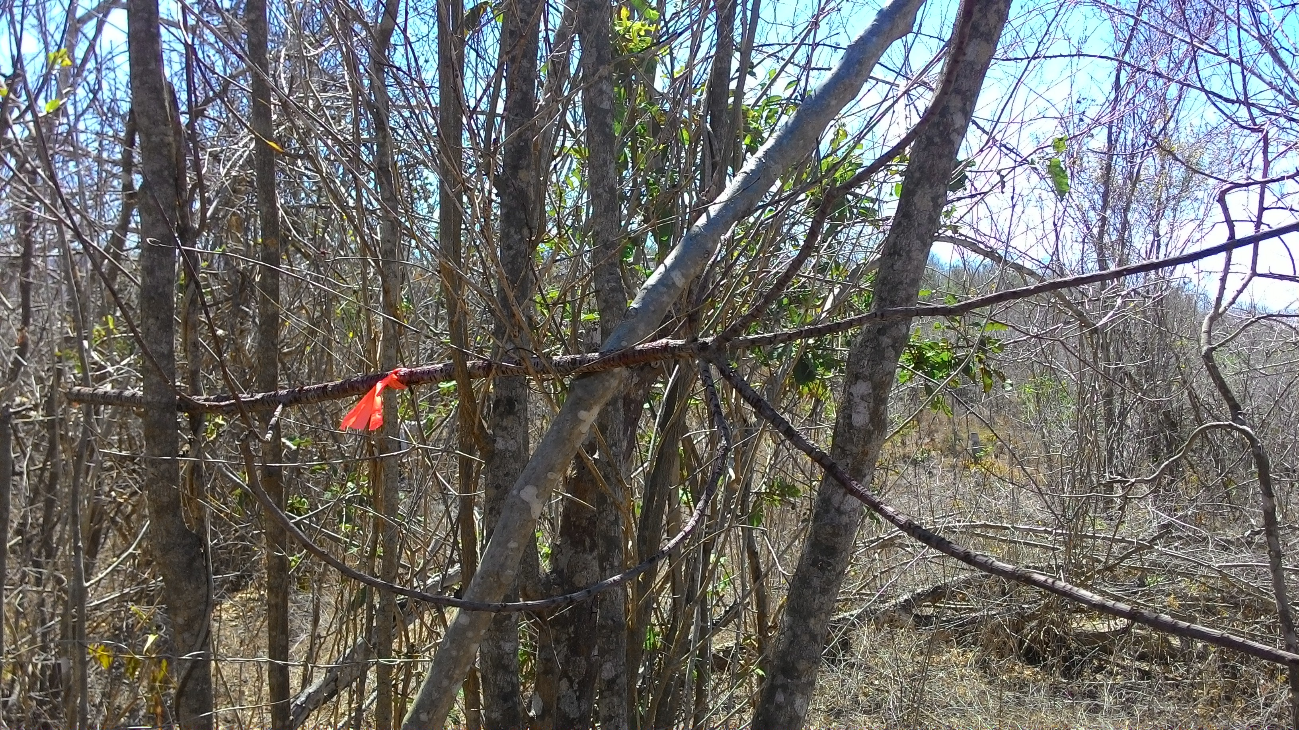


**Figure S1**. Example of a *Spondias purpurea* branch exposed to colonization by saproxylic beetles and their natural enemies in the understory of a tropical dry forest, in Jalisco, Mexico.


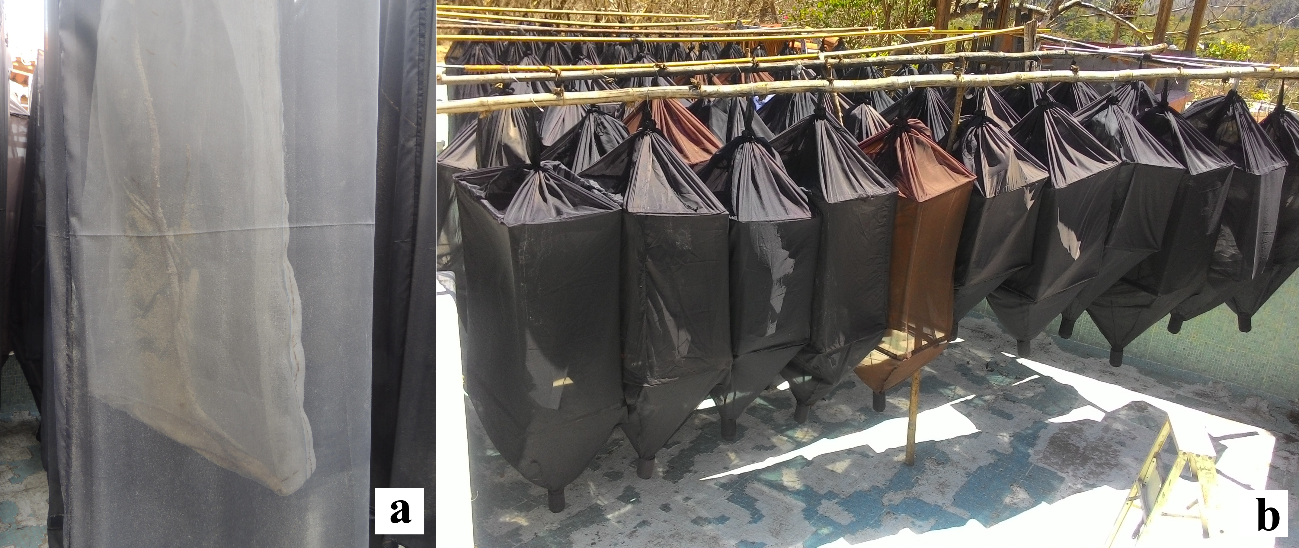


**Figure S2**. A branch of *Spondias purpurea* double-enclosed in mesh bags and emergence tulle traps (a). The traps were placed in an out-of-service swimming pool and maintained under local environmental conditions (b).

Tables

**Table S1**. Morphospecies richness (Rich.) and abundance (Abun.) of saproxylic beetles and predatory beetles that emerged from *Spondias purpurea* branches in a tropical dry forest, in Jalisco, Mexico, during two sampling years. Bostrichidae I refers to species with small body size (< 4,6 mm), while Bostrichidae II refers to species with large body size (> 4,6 mm). * Families of beetles considered predatory.

| Family: Subfamily | 2016 | | 2017 | | Total | | Body size |
| --- | --- | --- | --- | --- | --- | --- | --- |
|  | Rich. | Abun. | Rich. | Abun. | Rich. | Abun. | (mean) |
| Anthribidae | 5 | 19 | 2 | 6 | 7 | 25 | 3,06 |
| Bostrichidae I | 6 | 11003 | 5 | 1682 | 6 | 12685 | 3,90 |
| Bostrichidae II | 2 | 18 | 6 | 701 | 6 | 719 | 11,86 |
| Bostrichidae: Lyctinae | 2 | 92 | 2 | 2582 | 2 | 2674 | 2,78 |
| Bothrideridae* | — | — | 2 | 3 | 2 | 3 | 2,79 |
| Buprestidae | 2 | 13 | — | — | 2 | 13 | 5,35 |
| Carabidae* | — | — | 1 | 1 | 1 | 1 | 7,97 |
| Cerambycidae | 8 | 22 | 8 | 183 | 13 | 205 | 11,53 |
| Chrysomelidae: Bruchinae | 2 | 2 | — | — | 2 | 2 | 1,63 |
| Cleridae* | 4 | 8 | 4 | 9 | 5 | 17 | 4,22 |
| Corylophidae | 1 | 10 | — | — | 1 | 10 | 0,95 |
| Cryptophagidae | 1 | 1 | — | — | 1 | 1 | 2,02 |
| Curculionidae | 6 | 17 | 2 | 2 | 8 | 19 | 3,96 |
| Curculionidae: Scolytinae | 15 | 6983 | 10 | 1329 | 16 | 8312 | 1,91 |
| Dermestidae | 2 | 3 | — | — | 2 | 3 | 1,93 |
| Dytiscidae | 1 | 1 | — | — | 1 | 1 | 1,50 |
| Erotylidae | 1 | 8 | — | — | 1 | 8 | 1,38 |
| Histeridae* | 2 | 1542 | 2 | 4066 | 2 | 5608 | 2,06 |
| Hydraenidae | 1 | 5 | — | — | 1 | 5 | 1,42 |
| Hydrophilidae | 1 | 2 | — | — | 1 | 2 | 3,23 |
| Laemophloeidae | 3 | 25 | — | — | 3 | 25 | 1,87 |
| Latridiidae | 1 | 1 | — | — | 1 | 1 | 1,40 |
| Melyridae | 2 | 2 | 1 | 2 | 2 | 4 | † |
| Mycetophagidae | 1 | 1 | — | — | 1 | 1 | 2,26 |
| Passandridae* | 2 | 3 | 2 | 24 | 2 | 27 | 3,46 |
| Ptinidae: Anobiinae | 2 | 2 | — | — | 2 | 2 | 2,12 |
| Scarabaeidae | 2 | 3 | — | — | 2 | 3 | 8,72 |
| Scraptiidae | 1 | 1 | — | — | 1 | 1 | 1,82 |
| Silvanidae | 3 | 18 | — | — | 3 | 18 | 2,21 |
| Smicripidae | 1 | 13 | — | — | 1 | 13 | 1,24 |
| Staphylinidae* | 7 | 15 | — | — | 7 | 15 | 1,75 |
| Tenebrionidae | 3 | 20 | 6 | 101 | 7 | 121 | 4,05 |
| Trogossitidae* | 1 | 2 | 2 | 20 | 2 | 22 | 3,29 |
| Zopheridae | 2 | 19 | 1 | 1 | 2 | 20 | 3,57 |
| Total | 93 | 19874 | 56 | 10712 | 116 | 30586 |  |

† The few individuals were damaged by dermestids in the insect collection before measures were taken.

**Table S2**. Number of individuals of parasitoid morphospecies that emerged from *Spondias purpurea* branches in a tropical dry forest, in Jalisco, Mexico, during two sampling years.

| Family/subfamily/genus | |  | 2016 | 2017 | Total |
| --- | --- | --- | --- | --- | --- |
| Bethylidae |  | sp.1 | 63 | 4 | 67 |
|  |  | sp.2 | 1 | — | 1 |
|  |  | sp.3 | 2 | 3 | 5 |
|  |  | sp.4 | 2 | 2 | 4 |
|  |  | sp.5 | — | 2 | 2 |
|  |  | sp.6 | — | 3 | 3 |
| Braconidae | Brachistinae | sp.1 | 7 | — | 7 |
|  | Doryctinae | *Ecphylus* sp.1 | 261 | 1 | 262 |
|  |  | *Ecphylus* sp.2 | 750 | — | 750 |
|  |  | *Ecphylus* sp.3 | 16 | — | 16 |
|  |  | *Heterospilus* sp.1 | 30 | 10 | 40 |
|  |  | *Pambolidea* sp.1 | 3 | 10 | 13 |
|  |  | *Pambolidea* sp.2 | 5 | — | 5 |
|  |  | *Parallorhogas* sp.1 | 1114 | 93 | 1207 |
|  |  | sp.1 | 14 | 13 | 27 |
|  |  | sp.2 | 7 | — | 7 |
|  |  | sp.3 | — | 2 | 2 |
|  |  | *Whartonius* sp.1 | 24 | 1 | 25 |
|  | Euphorinae | *Cryptoxilos* sp.1 | 328 | 276 | 604 |
|  |  | *Cryptoxilos* sp.2 | 11 | 1 | 12 |
|  |  | *Cryptoxilos* sp.3 | 76 | 1 | 77 |
|  |  | *Cryptoxilos* sp.4 | 1 | — | 1 |
|  |  | *Cryptoxilos* sp.5 | 3 | — | 3 |
|  |  | sp.1 | 1 | — | 1 |
|  |  | sp.2 | — | 5 | 5 |
| Ceraphronidae |  | sp.1 | 3 | — | 3 |
|  |  | sp.2 | 1 | 1 | 2 |
|  |  | sp.3 | 1 | — | 1 |
|  |  | sp.4 | 1 | 1 | 2 |
| Chalcididae | Haltichellinae | sp.1 | 2 | — | 2 |
| Chalcidoidea |  | sp.1 | 4 | — | 4 |
|  |  | sp.2 | — | 1 | 1 |
| Encyrtidae |  | sp.1 | — | 11 | 11 |
| Eulophidae | Tetrastichinae | sp.1 | 10 | — | 10 |
| Eupelmidae |  | sp.1 | 1 | — | 1 |
| Eurytomidae |  | sp.1 | 98 | 5 | 103 |
|  |  | sp.2 | 24 | 1 | 25 |
| Platygastridae | Scelioninae | sp.1 | 10 | — | 10 |
|  |  | sp.2 | 3 | 1 | 4 |
|  |  | sp.3 | 1 | 2 | 3 |
| Pteromalidae |  | sp.1 | 1 | — | 1 |
|  |  | sp.2 | — | 2 | 2 |
|  | Ceinae | *Neocalosoter* sp.1 | 36 | 1 | 37 |
|  |  | *Neocalosoter* sp.2 | 6 | — | 6 |
|  | Cleonyminae | sp.1 | 1 | — | 1 |
| Total |  |  | 2922 | 453 | 3375 |
